# Molecular investigation of *Toxocara* infection from the serum of people living with HIV in Alborz, Iran

**DOI:** 10.1186/s12879-023-08250-8

**Published:** 2023-05-03

**Authors:** Mohammad Zibaei, Zahra Hatami, Farzaneh Firoozeh, Fatemeh Bakhshipour, Abolfazl Miahipour, Alireza Soleimani, Elham Shokri

**Affiliations:** 1 Department of Parasitology and Mycology, School of Medicine, Alborz University of Medical Sciences, P.O. Box: 3149779453, Karaj, Iran; 2 Department of Microbiology, School of Medicine, Alborz University of Medical Sciences, Karaj, Iran; 3 Department of Infectious Diseases, School of Medicine, Alborz University of Medical Sciences, Karaj, Iran; 4 Department of Parasitology and Mycology, School of Public Health, Tehran University of Medical Sciences, Tehran, Iran

**Keywords:** Toxocariasis, ELISA, PCR, People living with HIV, Zoonosis

## Abstract

**Background:**

*Toxocara* infection is one of the most common neglected infections of poverty and a helminthiasis of global importance. Traditional diagnostic methods such as antibodies detection in serum samples are limited due to cross-reactivity and poor sensitivity. The use of molecular base methods for diagnosis of *Toxocara* infection in Iran has not been fully explored. The purpose of the current study was to estimate the prevalence of *Toxocara* infection from serum samples of people living with HIV in Alborz province, Iran using serological and molecular methods.

**Methods:**

Blood samples were collected from 105 people living with HIV. Epidemiological data of participant were obtained through a structured questionnaire to investigate the risk factors. Patients CD4^+^ T cell count were recorded. Anti-*Toxocara* IgG antibodies were detected by ELISA, with a cut-off point of 11. PCR was performed to detect genetic material of *Toxocara* species in the serum samples.

**Results:**

The mean CD4^+^ count in HIV-infected individuals with positive toxocariasis serology was 255.1 ± 21.6 cells/µL. Seropositivity for *Toxocara* species was observed in 12/105 (11.4%) people living with HIV. Three samples gave positive results on PCR analysis. Based on the data, a statistically significant relationship was found between anti-*Toxocara* IgG antibodies seropositivity and underlying conditions (*p* = 0.017). No significant statistical association was observed between seropositivity for *Toxocara* and gender, age, exposure to domestic animals or pet keeping, education levels, and occupation (*p* > 0.05). The findings of PCR confirmed *Toxocara* DNA in 3/12 (25.0%) serum samples.

**Conclusion:**

These findings demonstrated for the first time that people living with HIV from Alborz province, are being exposed to this zoonosis and a relatively high seroprevalence of *Toxocara* in HIV/AIDS people needs comprehensive health education regarding personal hygiene and how to avoid exposure to this parasite infection, especially in people with an impaired immune system.

**Supplementary Information:**

The online version contains supplementary material available at 10.1186/s12879-023-08250-8.

## Background

*Toxocara* species is a common zoonotic parasite that infects humans and animals worldwide, with a prevalence ranging from 5.1 to 50.6% and 6.4–28.1% respectively [1]. Humans become infected most commonly by accidental ingestion of embryonated *Toxocara* species eggs present in contaminated food, water, or soil. Other routes of infection include consumption of undercooked infected organs of paratenic hosts containing encapsulated larvae [2]. In paratenic hosts like humans, larvae can migrate through the tissues and cause ocular larva migrans, visceral larva migrans, neurotoxocariasis, and covert and common toxocariasis. Finding a positive serological test result such as TES-ELISA is indicative of current or past infection in the affected population [3].

Despite several studies implicated *Toxocara* in various human visceral diseases, different causal agents share similar symptomatology. The pathogenic role of *Toxocara* is under debate, and several variables, like the *Toxocara* species, larval burden and host immune status, can affect the occurrence of the disease [4–6].

Toxocariasis is a neglected poverty infection that is underreported due to a lack of surveillance, even in resource-rich countries with strong health systems. The nonspecific, mild, or asymptomatic natural history of disease following infection can also contribute to this scenario. Developed countries, including the United States, Japan, Great Britain, France, Germany, and Italy, are world leaders in toxocariasis research, accounting for more than 34% of all published literature [7].

*Toxocara* infection is a common parasitic disease in tropical regions whose impact on public health is usually underestimated. The prevalence of human toxocariasis was estimated to be 38%, 34%, 24%, 23%, 11% and 8% in Africa, Southeast Asia, Western Pacific, Americas, Europe and Eastern Mediterranean region, respectively [8]. Most epidemiological studies on human toxocariasis in Iran have been carried out on children, and the seroprevalence ranged between 1.4 and 29.5%. This parasite is overrepresented in specific cultural groups and those with low socioeconomic status in Iran and appear to be emerging as a significant direct and/or food-borne infection [1].

The immunocompromised state of patients has increased susceptibility to a wide range of infections. Most reported agents are viruses, bacterial, fungal, and parasitic infections. For many years, researchers have debated the observation that helminth infections may increase susceptibility to HIV and that immune activation may have contributed to the spread of HIV in some regions of the world [9]. HIV infection causes various immunological changes, especially defects in cellular immunity and CD4^+^ T lymphocyte function. Although the prevalence of parasitic infections in the healthy population has decreased significantly in recent decades, the importance of helminthic infections in immunocompromised patients such as people living with HIV is of great importance, which can lead to complications and mortality [10]. In the United States, it is estimated that 20–47% of all people living with HIV develop active encephalitis due to the parasitic diseases that involve the brain, while the figures are 25 to 50% for Europe and Africa [11]. Because of the risk of damage to the central nervous system and neurotoxocariasis, and morbidity in these patients, we believe it is of utmost importance to find the prevalence of anti-*Toxocara* antibodies in HIV/AIDS patients.

Up to now, no study have been conducted on the prevalence of *Toxocara* infection among HIV/AIDS patients, in Iran. Therefore, the aim of this study was to determine the sero-molecular prevalence of toxocariasis in people living with HIV of Alborz province, Iran.

## Methods

### Ethics statement

The human research ethical approval for the study was granted by the Ethical Committee at the University of Medical Sciences in Alborz, and informed consent obtained from the participants prior to data collection (IR.ABZUMS.REC.1400.050). Written informed consent form was received from each study participant (for those under the age of 15 years, written informed consent form was received from their legal guardians).

### Study population

A cross-sectional study was conducted between September 2021 and January 2022 including people living with HIV followed at the Shahid Sabzeh Parvar Behavioral Diseases Counseling Center, Vice-Chancellor for Health at the Alborz University of Medical Sciences, Iran. A standardized questionnaire that was designed and applied during face-to-face interviews. The questionnaire included data about patients’ demographics (gender, age, educational levels, and job classification), CD4^+^ T cells count, potential risk factors for *Toxocara* infection (exposure to domestic animals and pet keeping), and detailed information on clinical characteristics and conditions such as diabetes, heart disease, central nervous system disorders, liver and kidney diseases. Patients were divided as to whether they were already on HAART or not.

### Collection of serum samples

Five milliliters of venous blood were drawn from each subject in a clean blood collection tube by trained medical laboratory technicians. Serum samples were separated by centrifuging at 3000 rpm for five min. The sera were collected on two mL tubes (Eppendorf) and stored at -20 °C until being used for the detection of anti-*Toxocara* IgG antibodies by ELISA and PCR.

#### ELISA

Anti-*Toxocara* antibodies were detected by commercial ELISA kit (NovaTec Immunodiagnostica GmbH, Dietzenbach, Germany) with modifications. In brief, sera diluted 1:100 with IgG sample diluent were applied (100 µL/well) in duplicates and incubated at 3 °C for 60 min. After washing three times in washing solution, the plates were incubated with 100 µL/well of *T. canis* protein A horseradish peroxidase-conjugate at room temperature for 30 min. For colour development, the plates were incubated with 100 µL of TMB substrate for 15 min at 37 °C. The reaction was halted (100 µL/well) with stop solution for 15 min. The final step was performed according to the manufacturer’s protocol, and the 450 nm was considered for the optical density (OD) reading of the examined wells (Microplate reader, STATFAX-2100-OHAHIO-USA). The cut-off value was between 9.0 and 11.0 NTU (NovaTec Unit), as recommended by the manufacturer’s calculation. Samples with NTU below ˂ 9.0 were negative; from 9.0 to 11.0 were in the grey zone, and ˃ 11.0 were positive.

#### PCR

Genomic DNA was extracted from serum samples using a DNA extraction kit (Circulating DNA isolation, DENAzist, Iran), according to the manufacturer’s instructions. PCR protocol and conditions were described by Zibaei et al. [12] and modified by Naderbandi et al. [13]. Briefly, PCR reaction was performed in Super master mix-2 × (12.5 µL), Primers (forward and reverse) (1 µL, 10 pmol/µL), DNA template (5 µL), DW (5.5 µL). The primers target fragments of about 330 and 600 bp from the *T. canis* and *T. cati*, respectively. The forward primer YY1 (5-CGGTGAGCTATGCTGGTGTG-3) for *T. canis* was used with the conserved reverse primer NC2 to amplify partial ITS-2. Similarly, for *T. cati*, the specific forward primer JW4 (5-ACTGTCGAGGATGAGCGTGA-3) was used with NC2 to amplify partial ITS-1, complete 5.8 S and ITS-2 rDNA. The PCR reaction conditions were: one cycle of primary denaturation (95 °C for 5 min), followed by 35 cycles of denaturation (94 °C for 45 s), annealing (58 °C for 35 s), extension (72 °C for 35 s), and one cycle of final elongation step (72 °C for 10 min). Amplified products were separated by electrophoresis on a 1.5% agarose gel and stained with 0.5 µg/mL ethidium bromide solution. A 100-bp ladder was used as DNA for estimating the size of the amplicons and photographed using a gel documentation system (UV Transilluminator, QUANTUM SD4-1000, VILBER, France).

### Statistical analyses

Continuous variables were summarized as mean ± standard deviation and categorical data as counts and percentages. Comparisons between groups were performed using chi-squared test or Fisher’s exact test for categorical variables, and t-test or Mann-Whitney test for continuous variables. The significance level for all analyses was set at *p* < 0.05. Data were analysed using SPSS, version 18.0 (SPSS Inc., Chicago, USA).

## Results

A total of 105 people living with HIV were enrolled in the study between September 2021 and January 2022. Fifteen samples were excluded from the molecular testing arm due to insufficient sample volume or sample processing deficiency, whose serological results were negative. The age range of the patients was between 20 and 71 years with a mean age of 42.1 ± 2.3 years. In the present study, 67.6% (71/105) the participants were in the age range of 21–49 years. A total of 47 (44.8%) patients were already on HAART and 58 (55.2%) started HAART after being participated in the study. Demographic and epidemiological data of the study population are described in Table 1. *Toxocara* positive subjects with underlying conditions compared to people without underlying diseases more frequently reported (66.7% vs. 33.3%, *p* = 0.017). Neither demographic characteristics such as gender, age and educational levels nor other epidemiological risk factors such as occupational, HAART or, pet keeping differed significantly between the *Toxocara* positive and *Toxocara* negative patients.

**Table 1:** Sociodemographic characteristics of the enrolled patients (*N* = 105)

Variable	*Toxocara* seropositive			*Toxocara* seronegative			Total	Statistical *(p*-value)
	N	%		N	%			
**Age Categories** (years)								
˂10	0	0.0		1	1.1		1	0.225
11–20	0	0.0		4	4.3		4	
21–30	2	16.7		10	10.8		12	
31–40	3	25.0		22	23.6		25	
41–50	5	41.7		30	32.2		35	
51–60	1	8.3		17	18.3		2	
˃60	1	8.3		9	9.7		2	
**Gender**								
Female	5	41.7		36	38.7		41	0.695
Male	7	58.3		57	61.3		64	
**Educational levels**								
No School	0	0.0		3	3.2		3	0.271
Some high School	7	58.3		54	58.1		61	
High School	2	16.7		22	23.6		24	
Some college/Graduate school	3	25.0		14	15.1		17	
**Occupation**								
Unemployed	3	25.0		23	24.7		26	0.091
Worker	2	16.7		5	5.4		7	
Housekeeper	3	25.0		27	29.0		30	
Other	4	33.3		38	40.9		42	
**Underlying conditions** ^**†**^								
Yes	8	66.7		76	81.7		84	0.017^**†††**^
No	4	33.3		17	18.3		21	
**Pet keeping**								
Yes	3	25.0		14	15.1		17	0.389
No	9	75.0		79	84.9		88	
**HAART** ^**††**^								
With HAART	5	41.7		42	45.2		47	0.578
Without HAART	7	58.3		51	54.8		58	

^**†**^ Underlying conditions include diabetes, heart disease, central nervous system disorders, and liver and kidney diseases; ^††^ Highly active antiretroviral therapy; ^††**†**^Statistically significant at 95% confidence intervals (*p* ˂ 0.05)

The mean CD4^+^ count in HIV/AIDS patients with positive toxocariasis serology was 255.1 ± 21.6 cells/µL. D4^+^ counts (cells/µL) were considered and categorized into three groups: CD4^+^ < 200, CD4^+^ 200–500, and CD4^+^ > 500. The seroprevalence of *Toxocara* infection in HIV/AIDS patients with CD4^+^ < 200 was 33.3% (4/12) and for the other groups were 50.0% (6/12) in 200 < CD4^+^ < 500, and 16.7% (2/12) in CD4^+^ > 500 (Table 2).

**Table 2:** Demographic, immunologic, and clinical manifestation of seropositive patients (*N* = 10)

Patient number	Age	Sex	Cut-off point^*^ (NTU)	CD4^+^ Count (cells/µL)	Clinical signs
# 10	45	F	19.0	486	Asymptomatic
# 23	54	M	40.5	113	cerebral palsy, Diarrhea^**^
# 28	46	M	16.9	472	Asymptomatic
# 32	28	F	21.2	190	Renal Failure, Diarrhea ^**^
# 36	37	M	11.5	812	Bronchopneumonia
# 39	27	M	11.7	460	Asymptomatic
# 43	60	M	24.8	142	Type 1 diabetes^**^
# 45	33	M	12.5	394	Hepatitis C
# 47	38	F	12.0	246	Epilepsy^**^
# 53	45	F	23.5	187	Tuberculosis, Thyroid cancer
# 56	48	M	18.5	365	Cardiovascular disease
# 100	40	M	12.7	495	Asymptomatic

^*^Cut- off point in ELISA: positive ˃ 11.0 NTU; NTU: NovaTec Unit; ^**^Significant results at 95% confidence intervals (*p* ˂ 0.05) in HIV/AIDS patients with underlying conditions

The ELISA method revealed that 12/105 samples (11.4%) were positive for anti-*Toxocara* IgG antibodies, 8/105 (7.6%) equivocal and inside the grey zone, while 85/105 (81.0%) were negative (Table 3).

**Table 3:** Distribution of anti-*Toxocara* IgG antibody titers determined by ELISA (*N* = 105)

	Patients N (%)	Titers (NTU^*^)	*p*-value
Anti-*Toxocara* IgG antibodies	85 (81.0)	< 9.0	0.484^**^
	8 (7.6)	9.0–11.0	
	12 (11.4)	> 11.0	

^*^NTU: NovaTec Unit, ^**^ No statistically significant at 95% confidence intervals

**Fig. 1:**
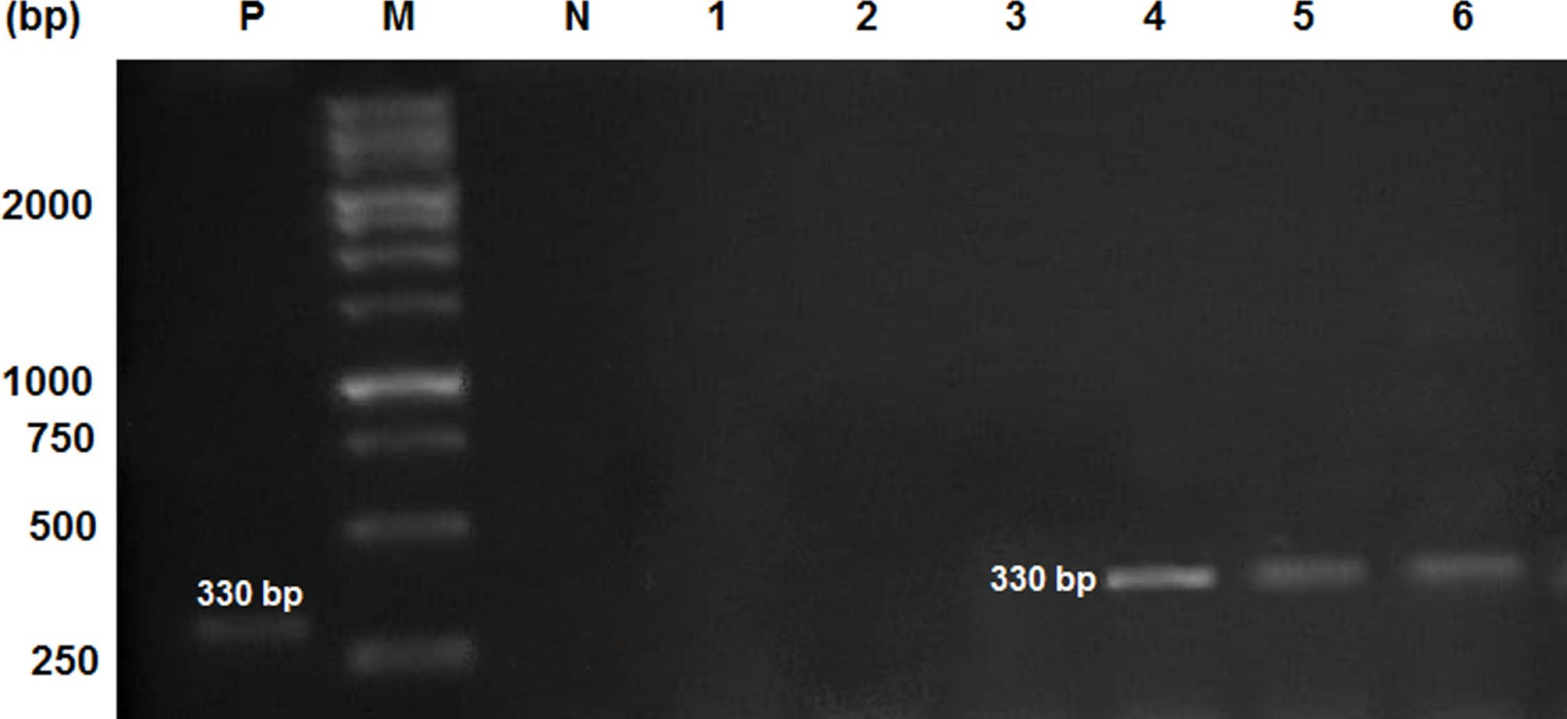
Analysis of PCR products amplified of *Toxocara* species from serum samples by 1.5% agarose gel electrophoresis. M: Molecular size marker 100-bp, P: Positive control with DNA of the parasite, N: Negative control without DNA. Lanes 4–6: *Toxocara canis* with YY1/NC2 primers. Full-length gel image is provided in Supplementary Fig. 1

The result of molecular detection of toxocariasis by ITS1 and ITS2 of *Toxocara* species in this study is shown in Fig. 1. The findings of PCR confirmed *Toxocara* DNA in 3/12 (25.0%) serum samples.

## Discussion

*Toxocara* is a zoonotic cosmopolitan infection that is more common in tropical areas with poor sanitation, where the soil is highly contaminated with *Toxocara* eggs from dogs and cats, and can survive for long time in moist soil [14]. Seroprevalence studies of *Toxocara* infection vary by geographic location. The global seroprevalence in general population is reported to be 38% [1]. In Iran, about 34.5% of the population is estimated to have antibodies against the infection [15]. The higher prevalence of anti-*Toxocara* IgG antibodies in more developed countries than Iran could be due to a higher consumption of raw or undercooked meat and contaminated vegetables. In addition, the transmission of toxocariasis is more common in regions with warm climates and lower altitudes than in regions with cold climates and mountainous areas such as European countries [16–18].

There are very few studies on the prevalence of toxocariasis in HIV/AIDS patients. In the present study, among the participants with HIV, 11.4% were seropositive for *Toxocara* infection, but this difference did not reach statistical significance. The prevalence of toxocariasis in our study was higher than that reported in a study of HIV-1 infected individuals in Beira, Mozambique with a prevalence of 7.3% [19]. In South Korea, it was shown in a report that the presence of toxocariasis cannot rule out HIV infection, and co-infection should be considered [20].

Our data demonstrated that there is no significant association between the prevalence of *Toxocara* infection and demographic and baseline characteristics such as exposure to domestic animals and pet keeping, gender, occupation, educational levels, and age groups in people living with HIV.

The findings of the present study showed that HIV/AIDS patients with underlying conditions was significantly associated with *Toxocara* infection (*p* = 0.017). Chronic medical conditions, what many may consider “underlying conditions”, include diabetes, heart disease, obesity, cancer, and kidney disease. Underlying diseases makes people more vulnerable to the infection [21]. In our study, eight cases (66.7%) of all *Toxocara* seropositive people had underlying conditions and clinical signs with a mean CD4^+^ count of 173.8 cells/µL. This suggests that the risk of infection is significantly increased in the patients with low CD4^+^ cell counts. *Toxocara* infection is a chronic condition that persists in the body for a long time in the absence of treatment, although it is not associated with immunosuppression because it is not opportunistic pathogen. Severe diarrhea is one of the most common clinical symptoms in people living with HIV and for treatment are usually prescribed cotrimoxazole, mebendazole or albendazole. However, the effect of the mentioned drugs on the results of the studies conducted in people living with HIV has not been determined [19].

It is estimated that approximately 1.5 billion or 24% of the world’s population are infected with parasitic helminths [22]. A widespread overlap between HIV and parasitic helminths has been reported in some regions of the world [23]. The prevalence of parasitic infections in people living with HIV could be explained by the fact that people with an impaired immune system are more susceptible to the infections [24]. In a study, it was shown that the overlap of helminthic and HIV infection may exacerbate anemia, because both infections are associated with nutritional deficiencies, leading to complex manifestations (HIV, helminths, and malnutrition). HIV infection impairs the individual’s metabolic ability to absorb, store, and use nutrients, resulting in nutrient deficiencies, compromising immunity and increasing the risk of infectious diseases. In addition, HIV-helminth coinfected individuals may lead to bone marrow failure, which reduces the production of red blood cells, leading to anemia [22].

We have found no significant association between CD4^+^ cell count and positive *Toxocara* serology in population living with HIV in the present study. The seroprevalence of *Toxocara* infection was higher in HIV/AIDS patients with between 200 and 500 CD4^+^/µL than in patients with more than 500 or less than 200. This could possibly be due to that although all groups are equally susceptible to toxocariasis, هn patients with CD4^+^ more than 500 cells/µL immune system is not yet compromised thus they have more ability to clear out the parasites before they become established in the body. On the other hand, in the case of patients with CD4^+^ counts less than 200 cells/µL are severely immunocompromised due to the loss of the ability to produce antibodies or to produce them at minimal levels undetectable by our assay. A serological study has demonstrated that the prevalence of toxocariasis was higher (although not significant) in HIV1^+^ patients with CD4^+^ count between 200 and 500 cells/µL [19].

In our study, before highly active antiretroviral therapy, the risk of toxocariasis was high in seropositive people living with HIV. This may be due of administration of chemoprophylactic regimen and HAART. No significant relationship was observed between seroprevalence of toxocariasis and highly active antiretroviral therapy (*p* = 0.578).

The results obtained for amplification of the ITS1 and ITS2 by PCR showed that 25.0% (3 out of 12) of samples were positive for *T. canis* in the people who were seropositive for anti-*Toxocara* antibodies. There are few studies were conducted to estimate the sero-molecular prevalence of parasitic infection in people living with HIV [25, 26]. To the best knowledge of the authors, the current study is one of the first study to present molecular detection of *Toxocara* species infection among people living with HIV. Based on a study, it has been found that ELISA could be detect toxocariasis in people living with HIV, but a clear understanding of the prevalence and manifestations of these co-infections, how best to diagnose subclinical cases, and how to manage diseases with concomitant antiretroviral therapy is needed [19].

## Limitations

The current study faced limitations such as resource constraints and lack of access to sequencing tests. Molecular testing with high sensitivity/specificity could be performed to overcome some limitations such as cross-reactivity.

## Conclusion

To conclude, this study was conducted considering the lack of information about toxocariasis from the subjects studied, to our knowledge this is the first study to molecular investigation of *Toxocara* in people living with HIV. The findings of the present study suggest that *Toxocara* is common in such patients (11.4%) and existence of underlying conditions or diseases increases the risk of toxocariasis in HIV/AIDS patients. It is recommended that more blood/serum samples from individuals living with HIV be screened and analyzed using molecular-based techniques as confirmatory tests.

## Electronic supplementary material

Below is the link to the electronic supplementary material.

Supplementary Material 1

Supplementary Material 2

## Data Availability

All the data generated or analyzed in this study are included in this published article. The datasets used and/or analyzed during the current study are available from the corresponding author on reasonable request.

## Abbreviations

AIDS: Acquired immunodeficiency syndrome
CD4: Cluster of differentiation 4
DNA: Deoxyribonucleic acid
DW: Distilled water
ELISA: Enzyme-linked immunosorbent assay
HAART: Highly active antiretroviral therapy
HIV: Human immunodeficiency virus
ITS: Internal transcribed spacer
OD: Optical density
PCR: Polymerase chain reaction
SPSS: Statistical package for the social sciences
TES: *Toxocara* excretory secretory
TMB: Tetramethylbenzidine
*T. canis*: *Toxocara canis*
*T. cati*: *Toxocara cati*.
