# Supplementary material for: Molecular investigation of Toxocara infection from the serum of people living with HIV in Alborz, Iran
Source: BMC Infect Dis. 2023 May 3;23:275. doi: 10.1186/s12879-023-08250-8 (PMC10155370; doi:10.1186/s12879-023-08250-8)
Supplement: Supplementary file 1 — Supplementary Material 1 [file 12879_2023_8250_MOESM1_ESM.doc]

**
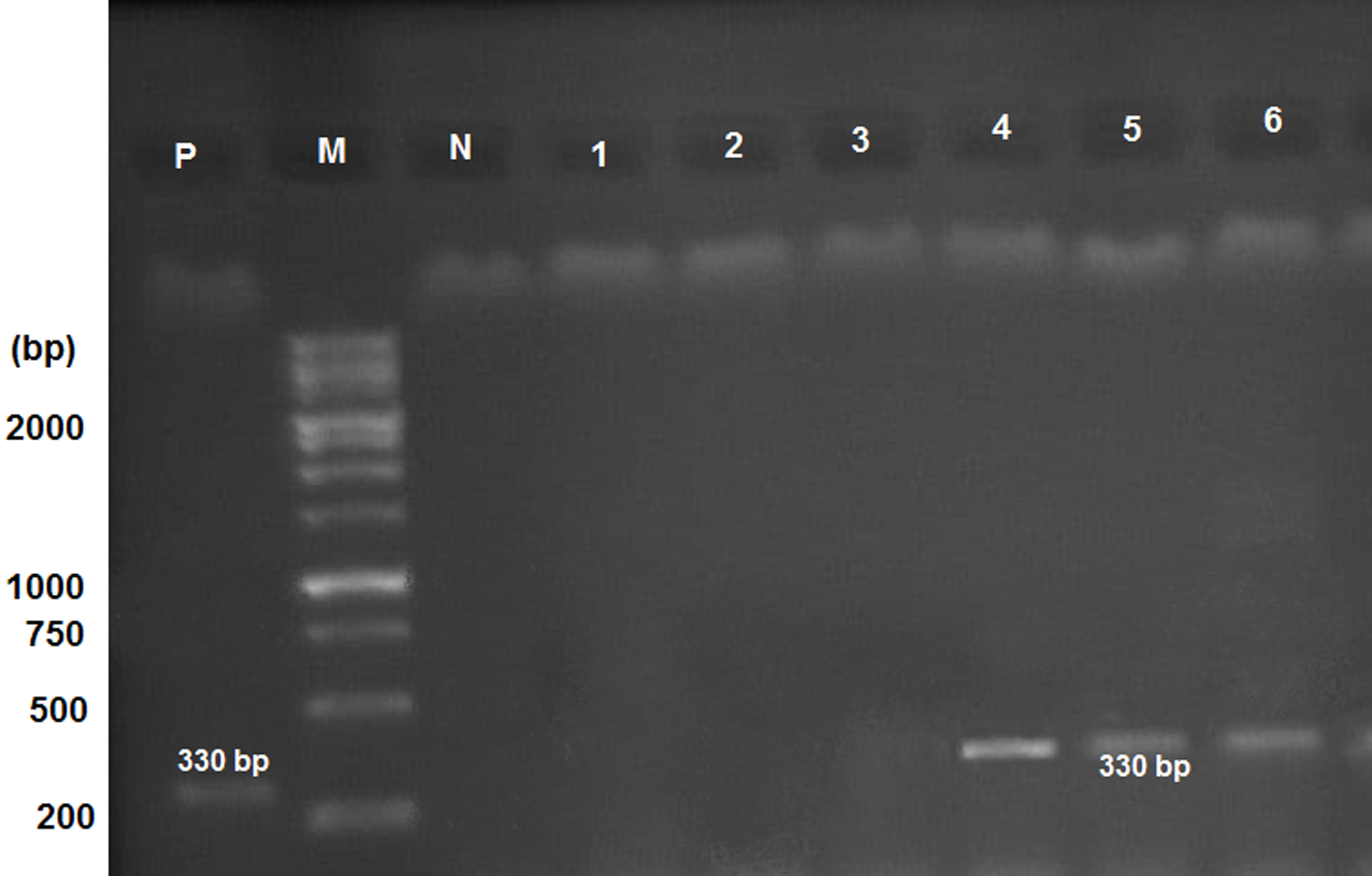
**

**Additional file 1**

Supplementary Fig. 1. Full-length gel images of PCR assay.

Analysis of PCR products of *Toxocara* species from serum samples by electrophoresis on 1.5% agarose gels. Figure shows the HIV-positive patients with *T. canis*. The infected subjects had *Toxocara* DNA amplification of 330 bp (lanes 4-6), patients #23, #32 and #43. Negative control (N) without DNA, 100-bp molecular size marker (M), and positive control (P) with DNA of the parasite (Adult worms).

**Additional file 2**

STROBE Statement Checklist of items that should be included in reports of cross-sectional studies.
