# Supplementary material for: Molecular investigation of Toxocara infection from the serum of people living with HIV in Alborz, Iran
Source: BMC Infect Dis. 2023 May 3;23:275. doi: 10.1186/s12879-023-08250-8 (PMC10155370; doi:10.1186/s12879-023-08250-8)
Supplement: Supplementary file 2 — Supplementary Material 2 [file 12879_2023_8250_MOESM2_ESM.doc]

STROBE Statement—Checklist of items that should be included in reports of ***cross-sectional studies***

|  | Item No | Recommendation |
| --- | --- | --- |
| **Title and abstract** | 1 | (*a*) Molecular investigation of *Toxocara* infection from serum of HIV-positive patients in Alborz, Iran |
|  |  | (*b*) **Background:** *Toxocara* infection is one of worldwide most common neglected infections of poverty and a helminthiasis of global importance. Traditional diagnostic technique such as antibodies detection in serum samples is limited by resources and poor sensitivity. The use of molecular base methods for diagnosis of *Toxocara* infection in Iran has not been fully explored. The purpose of the current study was to estimate the prevalence of *Toxocara* infection from blood of HIV-positive patients in Alborz, Iran using serological and molecular methods.  **Methods:** One hundred -five HIV-positive patients were subjected to a complete clinical exam, and blood samples were collected. Epidemiological data of participant were obtained through a structured questionnaire to investigate the risk factors. Anti-*Toxocara* IgG antibodies were detected by ELISA, with a cut-off point of 10. PCR was performed to detect genetic material of *Toxocara* species in the serum samples.  **Results:** Seropositivity for *Toxocara* species was observed in 12/105 (11.4%) HIV-positive patients. Two samples gave positive results on PCR analysis. Based on these data, a statistically significant relationship was found between anti-*Toxocara* IgG antibody seropositivity and underlying diseases (*p* = 0.017). No significant statistical association was observed between seropositivity for *Toxocara* and sex, age, exposure to domestic animals, soil contact, and diet (*P* > 0.05). The findings of PCR confirmed *Toxocara* DNA in 2/12 (16.7%) samples.  **Conclusion:** These findings demonstrated for the first time that HIV-positive patients from Alborz province, are being exposed to this zoonosis and a relatively high seroprevalence of *Toxocara* in HIV/AIDS patients needs comprehensive health education regarding personal hygiene and how to avoid exposure to this parasite infection, especially in people with a weak immune system. |
| Introduction | | |
| Background/rationale | 2 | *Toxocara* species is a common zoonotic parasite distributed worldwide infecting humans and animals, with a prevalence from 5.1–50.6% and 6.4–28.1% respectively [1]. Humans acquire this infection through accidental ingestion of infective *Toxocara* eggs in contaminated food or water, which is the primary route of infection. Other routes of infection include consumption of undercooked infected organs of paratenic hosts containing encapsulated larvae. In the paratenic hosts including humans, the larvae can migrate through the tissues and cause ocular larva migrans, visceral larva migrans, neurological syndrome, and hidden toxocariasis [2].  Despite several studies reported *Toxocara* implicated in different visceral diseases, potential pathogenetic factors have been described and its presence is frequently associated with symptoms in humans, its pathogenetic role is so far under debated and several variables, as well as the *Toxocara* species and load and host’s immune status could affect the occurrence of the disease [3-5].  *Toxocara* infection is a neglected infections of poverty that is underreported due to lack of surveillance, even in resource-rich countries with robust health systems. It is also underdiagnosed, due predominantly to the nonspecific, mild, or asymptomatic natural history of disease following infection. This parasite is overrepresented in specific cultural groups and those with low socioeconomic status in Iran and appear to be emerging as significant direct and/or food-borne infections [1]. The incidence of *Toxocara* in patients depends mainly on the existence of latent anti-*Toxocara* antibodies in the affected population [6]. Serological studies have demonstrated that 3.9% to 84.6% of people in Asia and 9.3% of people in Iranian communities show *Toxocara* infection [1]. In the United States, it is estimated that 20% to 47% of all patients with HIV develop inflammation of the active tissues of the brain due to the parasitic diseases while the figures are 25% to 50% for Europe and Africa [7]. Because of the risk of damage to the CNS and neurotoxocariasis, and morbidity in these patients, we believe it is of utmost importance to find out the prevalence of anti-*Toxocara* antibodies in HIV/AIDS patients. |
| Objectives | 3 | Up to now, no studies have been provided on the prevalence of *Toxocara* infection among HIV-positive people in Iran. Therefore, the objective of the current study was to determine the sero-molecular prevalence of toxocariasis among HIV-positive patients in Alborz province, Iran. |
| Methods | | |
| Study design | 4 | A cross-sectional study was conducted between September 2021 and January 2022 including HIV-positive patients followed at the Shahid Sabzeh Parvar Behavioral Diseases Counseling Center, Vice-Chancellor for Health at the Alborz University of Medical Sciences, Iran. A standardized questionnaire face-to-face interview was designed including demographic data (gender, age, educational levels, and job classification) and potential risk factors for *Toxocara* infection (exposure to domestic animals, soil contact, diet) as well as detailed information on clinical characteristics and underlying diseases from each participating subject (asthma, cerebrovascular diseases, chronic lungs and liver infection, and diabetes mellitus type 1 and 2). |
| Setting | 5 | Five millilitres of venous blood was drawn from each subject, clean blood collection tube by trained medical laboratory technicians. Serum samples were separated by centrifuging at 3000 rpm for 5 min. The sera were collected with Eppendorf tubes and stored at -20°C until being used for the detection of anti-*Toxocara* IgG antibodies by ELISA and PCR. |
| Participants | 6 | (*a*) HIV-positive patients referred to Shahid Sabzeh Parvar Behavioral Diseases Counseling Center, Alborz University of Medical Sciences, Iran. |
| Variables | 7 | Not applicable |
| Data sources/ measurement | 8* | Experimental design and protocol  One hundred-five HIV-positive patients were subjected to a complete clinical exam, and blood samples were collected  Briefly,  ELISA: Anti-*Toxocara* antibodies were detected by commercial Enzyme-Linked Immunosorbent Assay kit (NovaTec Immunodiagnostica GmbH, Dietzenbach, Germany), according to the manufacturer’s instructions. Briefly, serum samples diluted 1:100 with IgG sample diluent were applied (100 μL/well) in duplicates and incubated at 3 °C for 60 min. After washing 3 times in washing solution, the plates were incubated with 100 μL/well of *T. canis* protein A horseradish peroxidase-conjugate at room temperature for 30 min. For colour development, the plates were incubated with 100 μL of TMB substrate for 15 min at 37 °C. The reaction was halted (100 μL/well) with stop solution for 15 min and OD was determined at 450/620 nm for each well in a microplate reader (STATFAX-2100-OHAHIO-USA). The absorbance values were between 0.150–1.30 for cut-off control, < 0.200 and < cut-off for negative controls and > cut-off for positive controls, as recommended by the manufacturer’s instructions.  PCR: Genomic DNA was extracted from serum samples using a DNA extraction kit (Circulating DNA isolation, DENAzist, Iran) and submitted to PCR amplification using primers, which target fragments of about 330 bp and 600 bp from the *T. canis* and *T. cati* respectively. The forward primer YY1 (5-CGGTGAGCTATGCTGGTGTG-3) for *T. canis* was used together with the conserved reverse primer NC2 to amplify partial ITS-2. Similarly, for *T. cati*, the specific forward primer JW4 (5-ACTGTCGAGGATGAGCGTGA-3) was used with NC2 to amplify partial ITS-1, complete 5.8S and ITS-2 rDNA. Following PCR protocol and conditions is described in a previous study [8]. Briefly, PCR reaction was performed in Super master mix-2X 12.5 μL (), Primers (forward and reverse) (10 pmol/μL) 1 μL, DNA template 5 μL, DW 5.5 μL. The PCR reaction conditions were: one cycle of primary denaturation (95°C for 5 min), followed by 35 cycles of denaturation (94 °C for 45 s, annealing (58°C for 35 s, extension (72°C for 35 s), and one cycle of final elongation step (72°C for 10 min). Amplified products were separated by electrophoresis on a 1.5 % agarose gel and stained with ethidium bromide with 0.5 µg/mL ethidium bromide solution, and a 10 kbp ladder was used as DNA sizemarker for estimating the size of the amplicons and photographed using a gel documentation system (UV Transilluminator, QUANTUM SD4-1000, VILBER, France). |
| Bias | 9 | Describe any efforts to address potential sources of bias |
| Study size | 10 | Explain how the study size was arrived at |
| Quantitative variables | 11 | Explain how quantitative variables were handled in the analyses. If applicable, describe which groupings were chosen and why |
| Statistical methods | 12 | (*a*) Continuous variables were summarized as mean ± standard deviation and categorical data as counts and percentages. Comparisons between groups were performed using chi-squared test or Fisher’s exact test for categorical variables, and t-test or Mann-Whitney test for continuous variables. The significance level for all analyses was set at *p* < 0.05. Data were analysed using SPSS, version 18.0 (SPSS Inc., Chicago, USA). |
|  |  | (*b*) Describe any methods used to examine subgroups and interactions |
|  |  | (*c*) Explain how missing data were addressed |
|  |  | (*d*) If applicable, describe analytical methods taking account of sampling strategy |
|  |  | (*e*) Describe any sensitivity analyses |
| Results | | |
| Participants | 13* | (a) HIV-positive patients referred to Shahid Sabzeh Parvar Behavioral Diseases Counseling Center, Alborz University of Medical Sciences, Iran |
|  |  | (b) Give reasons for non-participation at each stage |
|  |  | (c) Consider use of a flow diagram |
| Descriptive data | 14* | (a) HIV-positive patients referred to Shahid Sabzeh Parvar Behavioral Diseases Counseling Center, Alborz University of Medical Sciences, Iran |
|  |  | (b) One hundred-five HIV-positive patients were subjected to a complete clinical exam, and blood samples were collected |
| Outcome data | 15* | Serum Samples from 105 patients was collected. Fifteen samples were excluded from the molecular testing arm due to insufficient sample volume or sample processing deficiency. The age range of the patients was between 20-71 years with mean age of 42.1 ± 2.3 years. More than half of the participants (71, 67.6%) were in the age range of 21-49 years.  The ELISA method revealed that 12/105 samples (11.4%) were positive for anti-*Toxocara* IgG antibodies, 8/105 (7.6%) equivocal, while 85/105 (81.0%) were negative. Considering the demographic variables, the frequency of anti-*Toxocara* IgG was significantly higher among HIV-positive patients with underlying diseases (*p* = 0.017). No statistically significant relationship was observed between PCR results, IgG antibodies’ seropositivity and other sociodemographic variables. Distributions of titters varying of the study population and clinical data of HIV-positive patients are described in Table 1 and Table 2.  The result of molecular detection of toxocariasis by ITS1 and ITS2 of *Toxocara* species in this study is shown in Figure 1. The findings of PCR confirmed *Toxocara* DNA in 2/12 (16.7%) serum samples. |
| Main results | 16 | (*a*) Give unadjusted estimates and, if applicable, confounder-adjusted estimates and their precision (eg, 95% confidence interval). Make clear which confounders were adjusted for and why they were included |
|  |  | (*b*) Report category boundaries when continuous variables were categorized |
|  |  | (*c*) If relevant, consider translating estimates of relative risk into absolute risk for a meaningful time period |
| Other analyses | 17 | Report other analyses done—eg analyses of subgroups and interactions, and sensitivity analyses |
| Discussion | | |
| Key results | 18 | Human toxocariasis is widespread, zoonotic cosmopolitan infection caused by the larval stages of *Toxocara* species, especially in people with immune deficiencies such as HIV/AIDS [9-11]. In Iran, screening and diagnosis of parasitic diseases in HIV-positive patients is based on parasitology, serology, and magnetic resonance imaging/computed tomography scans (MR/CT scans). When the CD4+ count is less than 50, in addition to antiretroviral the prophylaxis with anti-parasites drugs is performed.  In the current study, we reported a prevalence of 11.4% of anti-*Toxocara* IgG antibody positivity in HIV-positive patients. The present findings demonstrated a relatively higher anti-*Toxocara* IgG antibodies prevalence in HIV-positive patients compared with other studies. In a study conducted at the Ponta Gea Health Center in Mozambique, the seroprevalence of anti-*Toxocara* IgG antibody was 7.3% [9]. This diversity in findings could be explained by geographical differences, diagnostic techniques, public health status, eating habits and sample size.  Most epidemiological studies on human toxocariasis in Iran have been carried out on children and the seroprevalence ranged between 1.4% – 29.5% [1]. In a study conducted by the authors on people attending four educational and therapeutic centres in Alborz province, Iran, the seroprevalence of anti-*T*. *canis* IgG antibodies was 14.1% [12]. In general, the anti-*Toxocara* IgG prevalence in the studied people is higher in Iran compared with European countries (8.5-12.8%), while studies from Asia, Africa, and American countries reported prevalences of toxocariasis between 11.0-84.6%, 6.0–92.0%, and 19.7-26.0% respectively. The higher prevalence of anti-*Toxocara* IgG antibodies in more developed countries than Iran could be due to a higher consumption of raw or undercooked meat and contaminated vegetables. In addition, the transmission of toxocariasis is more common in regions with warm climates and lower altitudes than in regions with cold climates and mountainous areas such as European countries [13-15].  The results obtained for amplification of the ITS1 and ITS2 by PCR showed that 16.67 (2/12) of samples were positive for *T. canis* in the people who were seropositive for anti-*Toxocara* antibodies. To the best knowledge of the authors, the current study is one of the first study to present molecular detection of *Toxocara* species infection among HIV-positive patients. There are only a study reporting serological results of *Toxocara* in HIV-positive patients. In a research, it has been determined that ELISA could detect toxocariasis in HIV-positive patients, but highlighted that a clear understanding of the prevalence and manifestations of these coinfections, how best to diagnose subclinical cases, and how to manage diseases with concomitant antiretroviral therapy is needed |
| Limitations | 19 | The present study faced limitations such as resource constraints and lack of access to sequencing tests. Molecular testing with high sensitivity/specificity has been done to overcome some limitations. |
| Interpretation | 20 | Give a cautious overall interpretation of results considering objectives, limitations, multiplicity of analyses, results from similar studies, and other relevant evidence |
| Generalisability | 21 | Discuss the generalisability (external validity) of the study results |
| Other information | | |
| Funding | 22 | The authors state that they have not received any funding or grants to write and publish the results of this study. |

*Give information separately for exposed and unexposed groups.

**Note:** An Explanation and Elaboration article discusses each checklist item and gives methodological background and published examples of transparent reporting. The STROBE checklist is best used in conjunction with this article (freely available on the Web sites of PLoS Medicine at http://www.plosmedicine.org/, Annals of Internal Medicine at http://www.annals.org/, and Epidemiology at http://www.epidem.com/). Information on the STROBE Initiative is available at www.strobe-statement.org.
